# The Trunk Appearance Perception Scale (TAPS): a new tool to evaluate subjective impression of trunk deformity in patients with idiopathic scoliosis

**DOI:** 10.1186/1748-7161-5-6

**Published:** 2010-03-25

**Authors:** Juan Bago, Judith Sanchez-Raya, Francisco Javier Sanchez Perez-Grueso, Jose Maria Climent

**Affiliations:** 1 Spine Unit, Department of Orthopaedic Surgery, Hospital Vall d'Hebron, Universitat Autónoma de Barcelona, P° Vall d'Hebron, 119, 08035, Barcelona, Spain; 2 Department of Rehabilitation and Physical Medicine, Hospital Vall d'Hebron, Universitat Autónoma de Barcelona, P° Vall d'Hebron, 119, 08035, Barcelona, Spain

## Abstract

**Background:**

Outcome assessment in idiopathic scoliosis should probably include patients' perception of their trunk deformity in addition to self-image. This can be accomplished with the Walter Reed Visual Assessment Scale (WRVAS). Nevertheless, this instrument has some shortcomings: the drawings are abstract and some figures do not relate to the corresponding radiological deformity. These considerations prompted us to design the Trunk Appearance Perception Scale (TAPS).

**Methods:**

Patients with idiopathic scoliosis and no prior surgical treatment were included. Each patient completed the TAPS and SRS-22 questionnaire and underwent a complete radiographic study of the spine. The magnitude of the upper thoracic, main thoracic, and thoracolumbar/lumbar structural curves were recorded. The TAPS includes 3 sets of figures that depict the trunk from 3 viewpoints: looking toward the back, looking toward the head with the patient bending over and looking toward the front. Drawings are scored from 1 (greatest deformity) to 5 (smallest deformity), and a mean score is obtained.

**Results:**

A total of 186 patients (86% females), with a mean age of 17.8 years participated. The mean of the largest curve (CMAX) was 40.2°. The median of TAPS sum score was 3.6. The floor effect was 1.6% and ceiling effect 3.8%. Cronbach's alpha coefficient was 0.89; the ICC for the mean sum score was 0.92. Correlation coefficient of the TAPS mean sum and CMAX was -0.55 (*P *< 0.01). Correlation coefficients between TAPS mean sum score and SRS-22 scales were all statistically significant, ranging from 0.45 to 0.52 (*P *< 0.05).

**Conclusions:**

The TAPS is a valid instrument for evaluating the perception patients have of their trunk deformity. It shows excellent distribution of scores, internal consistency, and test-retest reliability, and has good capacity to differentiate the severity of the disease. It is simple and easy to complete and score, the figures are natural, and a new frontal view is included.

## Background

The perceived body image is an important factor in the assessment of health-related quality of life (HRQL) in persons with idiopathic scoliosis [1]; thus, self-image scales are included in the specific instruments used to evaluate these patients [2,3]. Nonetheless, these scales present some limitations. First, dissatisfaction with body image is common even in adolescents without scoliosis, since self-image partly depends on the perception of one's facial features and body mass [1]. Second, self-image scales show a significant, although only moderate, correlation with the radiologic magnitude of the curve [1-4]. This may indicate that other factors have an influence on the patient's view of the spinal deformity. Thus, the perception of body image and that of the trunk deformity would be complementary, but not equivalent.

To assess the outcome of any therapeutic intervention in this population, whether conservative or surgical, it would seem essential to measure the patients' perception of the trunk deformity in addition to their self-image, since the cosmetic disfigurement is one of their greatest concerns and a primary objective of treatment [5,6]. With this aim, Sanders et al. [7] developed the Walter Reed Visual Assessment Scale (WRVAS) and its extended version, the Spinal Appearance Questionnaire (SAQ) [8]. The WRVAS includes a series of figures representing 7 aspects of the deformity: spinal deformity, rib prominence, lumbar prominence, thoracic deformity, trunk imbalance, shoulder asymmetry, and scapular asymmetry. Each aspect is presented with 5 levels of increasing severity of the deformity. The SAQ, which is derived from the WRVAS, excludes the figure related to scapular asymmetry and introduces a side view of the body to assess spine prominence. The instrument includes other questions in which the patient scores several aspects of the cosmetic deformity as well as the surgical scar, and contains a total of 20 questions. Sanders et al. [7] confirmed that the instrument's metric properties (internal consistency, reliability, responsiveness, and validity) are adequate.

The WRVAS was evaluated by Pineda et al [9], who found that the scale has adequate internal consistency and significantly correlates with the magnitude of the curve and with the body image scale of the SRS-22 instrument. Nonetheless, Bago et al. [10] observed that some of the instrument's figures were not directly related to the corresponding radiological deformity. This lack of correlation was especially striking in the trunk imbalance and shoulder asymmetry items. These same investigators have additionally shown that elimination of these two questions (ie, leaving the WRVAS with 5 items) does not alter the metric properties of the scale [11].

The SRS-22 Questionnaire is well recognized and has become the most widely used patient-reported outcome instrument to evaluate the efficacy of several treatment regimens for idiopathic scoliosis. Despite the robustness of SRS-22, it has an inherent problem that affects its discriminant validity: a low correlation with the magnitude of the scoliosis [12,13]. Bago et al [11] demonstrated that this problem can be overcome by adding dimensions from other validated scales. Addition of the WRVAS to SRS-22 improved the coefficient of correlation with the Cobb angle.

The above-mentioned data indicate the usefulness of a figure-based scale such as the WRVAS for assessing patients' perception of their trunk deformity; however, the robustness of the instrument is based on its total score and not on the sub-scores for the various aspects of the deformity. Moreover, the WRVAS presents an image of the individual as seen from behind. In other words, the WRVAS measures how the patient feels that others see his or her back. Discussing the appearance of the WRVAS figures with our patients, we found that they consider them too abstract. These shortcomings and the patients' opinion prompted us to design the Trunk Appearance Perception Scale (TAPS) based on the WRVAS. This new scale has more realistic illustrations and a short-form format (3 images) that includes only the WRVAS figures corresponding to the 2 items that best correlate with the Cobb angle: the views of the trunk from the back and in the axial plane. The new feature of this scale is that it incorporates a frontal view, which we consider essential because it corresponds to what patients see when they look into a mirror, and this is probably the most realistic perception of one's body. The aim of the present study is to present this scale and its metric properties, in order to evaluate its possible use in determining scoliosis patients' subjective perception of their trunk deformity.

## Materials and methods

### Design and study population

This is a cross-sectional study, approved by the ethics committee for clinical research. Patients were recruited from 3 participating centers. The inclusion criteria were: a diagnosis of idiopathic scoliosis, age 10 to 40 years, no prior surgical treatment for this condition, magnitude of the main curve >10°, and informed consent to participate in the study. The sample size was calculated from data obtained in a previous study investigating the WRVAS scale [9], since we assumed that the mean scores of the WRVAS and the TAPS would be similar. The sample was stratified according to the magnitude of the major scoliotic curve (group 0, Cobb angle 10°-25°; group 1, Cobb angle 26°-45°; and group 2, Cobb angle ≥ 46°).

In the enrollment visit for the study, patients completed the measurement questionnaires and underwent a complete radiographic study of the spine in standing PA and lateral views. The magnitude of the upper thoracic, main thoracic, and thoracolumbar/lumbar curves were recorded following the method of Lenke [14]. To facilitate the statistical analysis, we established the variable, *maximum curve *(CMAX), defined as the curve showing the greatest magnitude among those presented by each patient.

### Measurement instruments

#### Trunk Appearance Perception Scale

**Figure 1:**
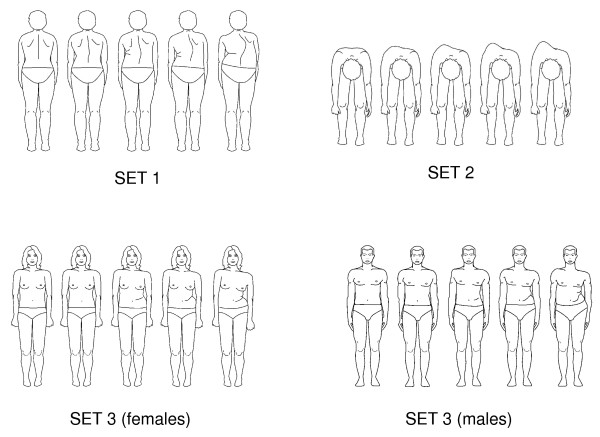
**Trunk Appearance Perception Scale (TAPS)**.

The TAPS (Figure 1) includes 3 sets of figures that depict the trunk from 3 viewpoints: looking toward the back, looking toward the head with the patient bending over (Adam's test), and looking toward the front. This last view has two sets of drawings, one for males and one for females. Each drawing is scored from 1 (greatest deformity) to 5 (smallest deformity) and a mean score is obtained by adding the scores for the 3 drawings and dividing by 3.

#### SRS-22 Patient Questionnaire

The SRS-22 contains 22 questions covering 5 domains: function/activity, 5 items; pain, 5 items; self-perceived body image, 5 items; mental health, 5 items; and satisfaction with treatment, 2 items. The satisfaction scale was not used in the present study. Each item is scored from 1 (worst) to 5 (best). In the present study, the results are expressed as the mean for each domain (total sum of the domain divided by the number of items answered) and the total score. The questionnaire used was the revised version, which includes a modification of question 18 [15,16].

#### Statistics

The statistical analysis was performed with the SPSS software, version 11.5. The validity study for the TAPS included the distribution of scores and determination of the floor effect (% of patients with the minimum score) and ceiling effect (% of patients with the maximum score). Internal consistency was examined with Cronbach's alpha coefficient. Test-retest reliability was determined with the intraclass correlation coefficient (ICC). Discriminant validity was evaluated by determining the correlation (non-parametric Spearman's correlation coefficient) between the total TAPS score and the largest curve (CMAX). Between-group comparisons were done with non-parametric tests. Convergent validity was assessed by analyzing the correlation (non-parametric Spearman correlation coefficient) between the total TAPS score and the total SRS-22 score, and more specifically, between the TAPS score and the score on the SRS-22 body image scale. Statistical significance for all tests was set at *P *< 0.05.

## Results

A total of 186 patients (160 females, 86%), with a mean age of 17.8 (± 6.4) years (range, 10-40), were included in the study. The mean magnitude of the upper thoracic curve was 35.6° (± 9.6); main thoracic curve 41.3° (± 18.1), and thoracolumbar curve 36° (± 17.8). At the time the assessment instruments were administered, patients were under conservative clinical and radiological monitoring, receiving orthotic treatment, or scheduled for surgery. The mean and SD of the CMAX for the total of patients and for each treatment group are shown in Table 1. In addition, the patients' descriptive data are presented according to the magnitude of the curve, age group, and sex. The scoliosis pattern, determined with the classification of Lenke [14], was as follows: 67 type 1, 11 type 2, 48 type 3, 6 type 4, 37 type 5, and 17 type 6.

**Table 1:** Magnitude of the largest curve for the total sample and by groups according to Cobb angle, treatment, age, and sex

	n (%)	CMAX average	SD
All patients	186	40.2°	18.7
			
Cobb angle 10°-25°	43 (23.1)	19.4°	4.4
Cobb angle 26°-45°	82 (44)	34.8°	6
Cobb angle ≥ 46°	61 (32.9)	62.1°	13.2
			
Observation	83 (44.6)	33.9°	17.8
Brace	59 (31.7)	32.8°	8.4
Surgery	44 (23.6)	61.9°	13.1
			
Age 10-19	150	37.4°	17.9
Age ≥ 20	36	51.8°	17.6
			
Females	160	41°	18.4°
Males	26	35.1°	19.6°

### Trunk Appearance Perception Scale

The median and interquartile range (IQR) for each of the 3 figures and the sum score and the percentage of patients with a minimum score (floor effect) and a maximum score (ceiling effect) are shown in Table 2. The median of the total score was 3.6 for the age group younger than 20, and 3.3 for patients aged 20 and older. This difference was statistically significant (Mann-Whitney, *P *= 0.001). In addition, the median of total score for females was 3.6 and for males 4, although the difference was not significant.

**Table 2:** Median (IQR), floor effect and ceiling effect for each figure, and the mean sum

TAPS	median (IQR)	Floor effect (%)	Ceiling effect (%)
1	3 (1)	8.1	7.0
2	4 (1)	1.6	17.7
3	3 (1)	5.4	9.1
Mean Sum	3.6 (1)	1.6	3.8

**Figure 2:**
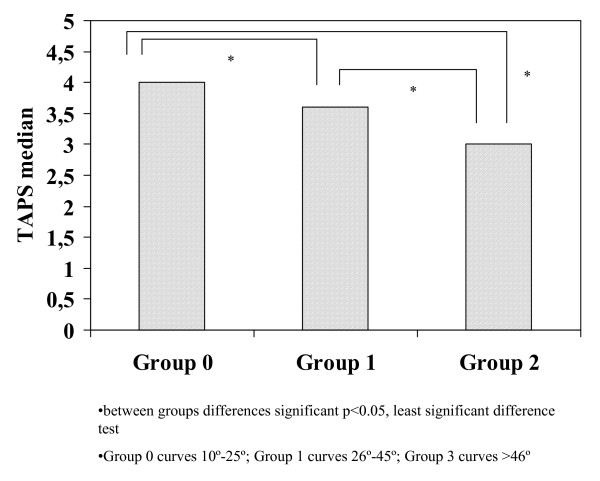
**Histogram of TAPS median for groups stratified according to maximum curve magnitude (Group 0 10°-25°, Group 1 26°-45°, Group 2 >46°)**.

Total score of the scale was significantly different between the groups stratified according to the CMAX value, with results of 4.0, 3.6, and 3, respectively (Kruskall-Wallis test, *P *= 0.0001) (Figure 2). No differences were found between the median total TAPS scores for the different types of Lenke curve patterns (Kruskal-Wallis test, *P *> 0.05).

Analysis of the influence of the type of treatment (observation, orthesis, or proposed surgery) on the total TAPS score revealed statistically significant differences between the 3 groups: 3.7, 3.6, and 2.7, respectively (Kruskall-Wallis, *P *= 0.0001). The post hoc analysis (least significant test) showed that patients in the group scheduled for surgery had a lower score than the other groups (*P *< 0.05). Nonetheless, these differences may be attributable to the fact that the magnitude of the curve was larger (*P *= 0.0001) in patients scheduled for surgery than in the group treated with orthotics or those under observation (Table 1).

The Cronbach alpha coefficient for the scale was 0.89, indicating excellent internal consistency. The alpha coefficient was similar in patients younger than 20 years (0.88) and those 20 and older (0.88), and was slightly higher in females (0.89) than in males (0.84).

### Test-retest Reliability

A random sample of 35 patients from one of the participating centers (HVH) completed the scale one week after the first response. The intraclass correlation coefficient (ICC) was calculated and yielded a value of 0.92. for the mean sum.

### SRS-22 Questionnaire

The mean total score (excluding satisfaction) was 4.08 ± 0.4, and the means of the separate scales were: pain 4.31 ± 0.6, function 4.46 ± 0.5, body image 3.47 ± 0.6, and mental health 4.0 ± 0.4.

### Discriminant Validity

The discriminant validity of the TAPS was determined by analyzing the correlation between the TAPS scores and the CMAX. The Spearman's correlation coefficients between the CMAX and the scores for the TAPS figures, TAPS total score, and the magnitude of the upper thoracic (UpTh), main thoracic (MTh), and thoracolumbar/lumbar (ThL) curves are shown in Table 3.

**Table 3:** Spearman correlation coefficients between each TAPS figure and the magnitudes of the curves

	UpTh	MTh	ThL	CMAX
TAP1	-.32	-.42**	-.65**	-.51**

TAP2	-.26	-.38**	-.50**	-.47**

TAP3	-.47	-.41**	-.58**	-.49**

Mean Sum	-.49*	-.44**	-.65**	-.55**

* p = 0.05 ** p < 0.01

Abbreviations: UpTh, upper thoracic; MTh, main thoracic, ThL, thoracolumbar/lumbar; CMAX, largest curve

### Convergent Validity

To determine the convergent validity of the TAPS, the correlation between the TAPS score and SRS-22 score was analyzed. Spearman's correlation coefficients are shown in Table 4. All were statistically significant (p < 0.01), although the SRS-22 scores showing the highest correlation with the TAPS were the self-perceived body image scale (range, 0.43-0.54) and the total score (range, 0.45-0.52.).

**Table 4:** Spearman correlation coefficients between the TAPS figures and the different SRS-22 scales

TAPS	SRS pain	SRS function	SRS image	SRS Mental health	SRS sum
1	0.30	0.26	0.51	0.28	0.47

2	0.34	0.23	0.43	0.33	0.47

3	0.36	0.21	0.50	0.24	0.45

Mean Sum	0.37	0.26	0.54	0.30	0.52

P ≤ 0.05 for all coefficients

## Discussion

The characteristics of the Trunk Appearance Perception Scale demonstrate that it is a valid instrument to evaluate the subjective perception of the trunk deformity in patients with idiopathic scoliosis. The floor and ceiling effects of the TAPS (1.6% and 3.8%, respectively) compare favorably with those of the WRVAS, in which the majority of the figures have a floor effect greater than 15% [9]. These data suggest that the TAPS may be sensitive to the changes that occur following a specific treatment. In the comparison of the mean WRVAS and TAPS scores, it should be remembered that the direction of the scoring is inverse: whereas the WRVAS scores run from best to worst, the TAPS scores run from worst to best. This was done so that the TAPS would be scored in the same way as the SRS-22 Questionnaire. The internal consistency of the TAPS (Cronbach's alpha coefficient 0.89) and test-retest reliability (ICC 0.92) are excellent and similar to those reported for the WRVAS [9] and SBQ [8]. The internal consistency is similar in males and females, and in patients younger than 20 and those 20 years or older.

TAPS score was similar in both sexes, but there was a significant difference according to age, with younger patients showing a higher score than adults. The TAPS score was also significantly different between the treatment groups. The correlation analyses seem to indicate that these differences are attributable to the effect of the magnitude of the curve, which was larger in the group of patients older than 20 and those receiving surgical treatment. We thus decided to stratify the sample according to the radiologic magnitude of the curve, and for this reason, there is an evident imbalance in the number of cases with respect to the age and sex groups. As regards the treatment, patients were classified into 3 large groups (observation, bracing, or surgery), but there was some heterogeneity in their composition. The observation group included patients who had never received any type of treatment and patients who had been treated with braces in the past. Within the bracing group, there were patients starting this treatment and others who would soon complete it. The number of hours patients used these devices was not recorded. Therefore, we cannot rule out an effect of these variations on the TAPS score.

The effect of the magnitude of the curve on the TAPS score is evident. TAPS shows a good correlation with the magnitude of scoliosis (rho = -0.55), although it is somewhat lower than has been reported for the WRVAS (r = 0.69) [9]. The correlation is, however, higher than that reported between the SRS-22 and the magnitude of the curve [12]. The TAPS can discriminate between curves that are generally considered candidates for surgical treatment (curves >45°, mean CMAX 62°) and those that can be treated by other means. Of course, it is beyond the scope of our objectives to discuss whether a Cobb angle of 45° determines the need for surgical treatment, but this is the threshold generally applied [17].

The only radiologic variable included in the present study was the Cobb angle. In contrast to the WRVAS analysis [10], neither the shoulder imbalance nor the position of the C7 plumbline was included. In the present developmental phase of the TAPS, we considered that to evaluate the discriminant validity of the instrument it was essential to analyze the correlation with the variable that best describes the severity of the disease: that is, the Cobb angle. Moreover, in light of the lack of relationship between the WRVAS and the trunk or shoulder imbalance, it was very possible that these variables would not have an influence on the TAPS score either. Nonetheless, we realize that this omission might be considered a limitation of the study because it makes a face-to-face comparison with the WRVAS difficult. Furthermore, data from the physical examination were not considered for the analysis. This is also related to our focus on evaluating the metric properties of the instrument in this phase of its development. We believe that the data from the physical examination (waist asymmetry, scapula asymmetry, shoulder level, rib hump) are less reliable than the Cobb angle and that is why only Cobb angle was used to analyze the discriminant capacity of the TAPS. These other measures could be of interest, however, for future investigation in the relationship between the TAPS and the clinical variables.

**Figure 3:**
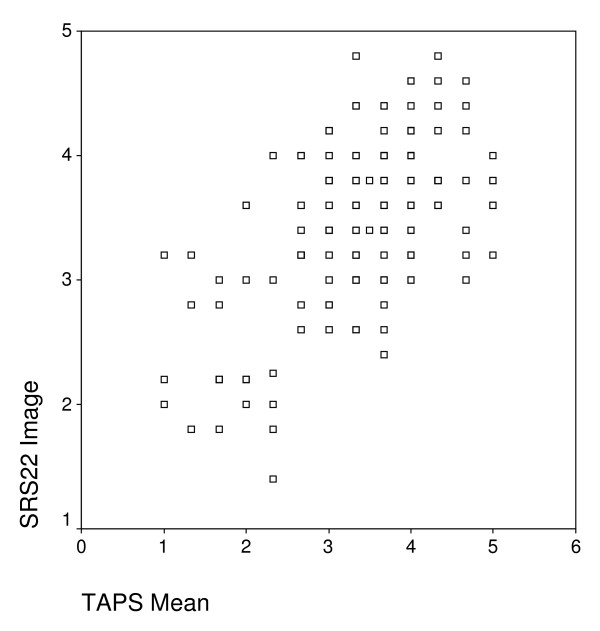
**Scatter plot between the SRS-22 self-image subscale and the mean score of TAPS**.

The TAPS score correlated significantly with the various SRS-22 subscales, although the highest correlations were obtained for the body image subscale and the total score. The correlations found between the TAPS and SRS-22 are similar to those of the WRVAS and SRS-22. Pineda et al [9] reported a correlation coefficient of 0.54 for the mean sum score (0.52 for TAPS) and 0.57 for the image subscale (0.54 for TAPS). Although the correlation of the TAPS with the SRS-22 Questionnaire was statistically significant, it is not extremely high (highest coefficient, 0.54). As can be seen in the scatter plot, there is considerable dispersion between the points plotted for the SRS-22 image subscale and the TAPS average score (Figure 3). This may indicate that although the two dimensions (body image and trunk deformity) belong to the same area of cosmesis, they evaluate somewhat different aspects and therefore, the two scales can be considered complementary.

It would be interesting to know the process that occurs for individuals with scoliosis to become aware of their deformity. We have the impression that various external factors play a part, such as the concern of third parties (particularly parents) or the image of the twisted spine on radiographs. We wonder to what degree the patients' scoring on the WRVAS or figures 1 and 2 of the TAPS (all views from behind) reflects the input they have received of how others see their trunk or the impression produced by the radiographs. This question led us to include a frontal view in the scale, this being the only direct view patients have of their torso. Of note, the TAPS scores showed a higher correlation with the magnitude of the thoracolumbar curve (rho = -0.65) than with the major thoracic curve (rho = -0.44). The fact that there was no correlation with the magnitude of the upper thoracic curve is also interesting. This may be because the thoracolumbar curve causes a more pronounced alteration of the waist symmetry, a feature that is very evident when looking in a mirror. This finding supports the appropriateness of including a frontal view in the scale, in contrast to the WRVAS (and SAQ), which only show the view from behind.

The WRVAS has shown excellent metric properties [7,9], but some of its figures (particularly shoulder asymmetry and trunk imbalance) do not relate to the corresponding radiological deformity. Nevertheless, drawings of the deformity in a coronal view and axial view have shown an excellent correlation with the Cobb angle [10]; hence it would seem logical to use them in an assessment instrument based on illustrations. In the informal discussions we had with patients about the appearance of the WRVAS figures, there were many comments indicating that the drawings did not seem realistic, and that patients did not identify with them. Based on these impressions, we decided to use more natural drawings, while maintaining simplicity of the lines. Keeping in mind the above-mentioned data, we designed the TAPS with drawings that are simple, but natural, and included only 3 views of the trunk: those that have shown the best qualities on the WRVAS, the posterior coronal view and axial view, and a new frontal view. We recognize that the TAPS was developed from the information obtained from an analysis of the WRVAS and consider it to be derived from that instrument.

As is the case of the WRVAS, the SAQ has satisfactory metric properties and a good capacity for differentiating disease severity. Because it includes a larger number of questions, the SAQ can provide more information, but it is not clear what use this additional information might have. It may be useful for decision making in individual cases, but its size (20 questions) may limit its application in routine practice. Short forms are commonly used in assessing HRQOL to facilitate completion and scoring. A future subject of research would be to determine the value of adding the TAPS as an additional dimension to the SRS-22 questionnaire. A recent study by Bagó et al. [11] analyzed the impact of adding the WRVAS as an additional scale to the SRS-22. The correlation coefficient with the magnitude of the curve was -0.37 for the SRS-22 alone and -0.52 with addition of the WRVAS, with no decrease in the internal consistency (Cronbach's alpha coefficient) of the instrument. We believe it is preferable to add a scale of 3 questions to the SRS-22 (25 questions in all) than a scale of 20 questions such as the SAQ (42 in all). Moreover, it is reasonable to infer that some of the SAQ drawings might have the same problems of validity as have been observed in the WRVAS [10].

TAPS is one step more in the effort to measure the trunk deformity from the patient's perspective. The dilemma of whether it is preferable to use the patient-reported subjective perception of the deformity or an objective measurement method to assess the deformity remains to be resolved. Objective methods, such as the ISIS system [18,19] and the Quantec system [20,21], are based on optoelectronic technology. These instruments are expensive, and reliable measurement depends on the position of the patient and the skill of the examiner. Asher et al. [20] reported that the measures obtained correlate weakly with the radiologic magnitude of the curve. Moreover, there was no correlation between the various SRS-22 dimensions and a comprehensive coronal plane surface topography measurement (POTSI, the posterior trunk symmetry index).

Another reported approach is assessment of severity by judges (physicians or others) who score several visible aspects of the deformity [19,22-26]. The interobserver correlations found are usually satisfactory (r>0.5), but the exact degree of agreement is generally low (kappa <0.4). Theologis et al [19] proposed a cosmetic spinal score performed by judges who score photographs (posterior, lateral, and forward bending) on a scale of 1 to 10. The correlation between this cosmetic score and the Cobb angle was 0.46. Donaldson et al. [23] studied the scores of 5 spine surgeons on clinical photographs assessing overall appearance on a scale of 1 to 5. The correlation with the Cobb angle was 0.53. These reported correlations are similar to those found between the TAPS and the Cobb angle (r = -0.55).

From a methodological perspective, it would be ideal that the patients' perception and the clinical and radiological measures of the deformity were highly correlated. In daily practice, however, it is common to encounter discrepancies between the radiologic deformity (Cobb angle) and the aesthetic deformity. This situation has ignited the debate as to what aspect, the radiologic or aesthetic problem, should be the primordial target of treatment. We believe it is crucial to know the patient's perspective in this debate, and the TAPS can be useful for this purpose.

In conclusion, the TAPS is a valid instrument for evaluating the perception patients have of their trunk deformity. The TAPS is based on the WRVAS, and its metric characteristics show better distribution of scores and similar internal consistency and test-retest reliability. The correlation with the magnitude of the curve is somewhat lower than the WRVAS, although the TAPS shows a good capacity to differentiate the severity of the disease. As to practical considerations, it is a simple scale that is easy to complete and score, the figures are more natural than those of the WRVAS, and a new frontal view is included, an element that has not been used previously.

## Competing interests

The authors declare that they have no competing interests.

## Authors' contributions

**JB **has made substantial contributions to conception and design, or acquisition of data, or analysis and interpretation of data; has been involved in drafting the manuscript or revising it critically for important intellectual content; and has given final approval of the version to be published.

**JS-R **has made substantial contributions to conception and design, or acquisition of data; and has given final approval of the version to be published.

**FJSP-G **has made substantial contributions to conception and design, or acquisition of data; and has given final approval of the version to be published.

**JMC **has made substantial contributions to conception and design, or acquisition of data, or analysis and interpretation of data; has been involved in drafting the manuscript or revising it critically for important intellectual content; and has given final approval of the version to be published.
